# Antimicrobial susceptibility profiles of *Mycoplasma hyosynoviae* strains isolated from five European countries between 2018 and 2023

**DOI:** 10.1038/s41598-024-85052-1

**Published:** 2025-01-07

**Authors:** Ulrich Klein, Dorottya Földi, Eszter Zsófia Nagy, Lilla Tóth, Nikolett Belecz, Karola Költő, Enikő Wehmann, Szilvia Marton, Marianna Merenda, Michele Gastaldelli, Salvatore Catania, Joachim Spergser, Ute Siesenop, Philip Vyt, Krisztián Bányai, Zsuzsa Kreizinger, Wouter Depondt, Miklós Gyuranecz

**Affiliations:** 1 Huvepharma NV, Antwerp, Belgium; 2 HUN-REN Veterinary Medical Research Institute, Budapest, Hungary; 3 National Laboratory of Infectious Animal Diseases, Antimicrobial Resistance, Veterinary Public Health and Food Chain Safety, Budapest, Hungary; 4 Mycoplasma Unit, Istituto Zooprofilattico Sperimentale Della Venezie, Buttapietra, VR Italy; 5 Institute of Microbiology, University of Veterinary Medicine, Vienna, Austria; 6 Institute for Microbiology, University of Veterinary Medicine Hannover, Hannover, Germany; 7 Dialab Diagnostic Laboratory, Belsele, Belgium; 8 University of Veterinary Medicine, Budapest, Hungary; 9 MolliScience Kft., Biatorbágy, Hungary

**Keywords:** *Mycoplasma hyosynoviae*, Swine, MIC, Broth micro-dilution, Antimicrobial resistance, Bacteriology

## Abstract

*Mycoplasma* (*M*.) *hyosynoviae* is a facultative pathogen, causing arthritis in finisher pigs world-wide. In the absence of a commercial vaccine improvement of housing conditions and antibiotic therapy are the only options to alleviate the clinical signs. This study aimed to determine antibiotic susceptibility profiles of 106 M*. hyosynoviae* isolates against ten antibiotics licensed for veterinary use in cases of arthritis. The isolates were collected between 2018 and 2023 from five European countries: Austria (n = 20), Belgium (n = 20), Germany (n = 25), Hungary (n = 21) and Italy (n = 20). The minimal inhibitory concentrations (MIC) were determined by broth micro-dilution assay. The tested isolates were highly susceptible to tiamulin (MIC_90_ ≤ 0.039 µg/ml), tylvalosin (MIC_90_ ≤ 0.039 µg/ml) and lincomycin (MIC_90_ ≤ 0.25 µg/ml). Low concentrations of tylosin (MIC_90_ 0.5 µg/ml) and tilmicosin (MIC_90_ 1 µg/ml) inhibited the growth of the isolates. While moderate minimal inhibitory concentrations were detected for doxycycline (MIC_90_ 0.312 µg/ml), oxytetracycline (MIC_90_ 2 µg/ml), enrofloxacin (MIC_90_ 0.625 µg/ml) and florfenicol (MIC_90_ 2 µg/ml), only high concentrations of tulathromycin (MIC_90_ 64 µg/ml) inhibited the growth of the isolates. Statistical analysis revealed significant differences between countries in case of enrofloxacin, where the Hungarian isolates showed the lowest MIC values, and the German isolates the highest MIC values among the tested countries. Our results show that European *M. hyosynoviae* isolates are generally susceptible to the tested antibiotics with the exception of tulathromycin. The country specific differences indicate the importance of regular susceptibility testing of isolates on a Pan-European level.

## Introduction

*Mycoplasma* (*M.*) *hyosynoviae* is a facultative pathogen in swine. This species is phylogenetically distant from other pathogenic swine mycoplasmas (*M. hyopneumoniae* and *M. hyorhinis*) and hydrolyses arginine as an energy source^1^.

Infections were reported from Australia, Canada, Denmark, England, Japan, Korea, the United States and Thailand, but *M. hyosynoviae* is presumed to be distributed worldwide^2,3^. *M. hyosynoviae* colonises the tonsils (mainly tonsilla palatina) where it may persist over a long period of time. The pathogen spreads via nasal secretions among animals. Colonisation rates of the tonsils are typically low at weaning and peak between 10 and 16 weeks of age^2,3^. The predisposing factors and triggers of hematogenous spread are not clear, but once *M. hyosynoviae* spreads from the tonsils it colonises the joints^2^. Clinical signs typically appear in animals older than 10 weeks of age (around 30–40 kg weight) and above 100 kg weight. The disease manifests in the swelling of the joints of the hind legs or lameness of the hind legs, which usually lasts for four to 5 days^2,4^. The organism may only be present transiently within the joints making diagnosis sometimes challenging^4^. Sub-clinical *M. hyosynoviae* infection can also occur as the bacterium can be isolated from joints and blood of apparently healthy pigs as well^4^. Similarly, colonisation of the tonsils is not in relation to the appearance of clinical infection, as only a portion of colonised pigs show clinical signs^3,5^. The incidence rates of arthritis caused by *M. hyosynoviae* in the United States increased around 2010^6^, however between 2017 and 2022 a decrease in the number of diagnosed cases was observed (although it should be noted that the number of tests also decreased in this period)^7^. In a recent report from a swine breeding region in Italy, similar trends were found as in the United States where *M. hyosynoviae* was only detected in a low number of arthritis cases^8^.

There are no commercial vaccines against *M. hyosynoviae* available, therefore the control and treatment of infection mainly relies on antibiotic treatment. Members of the *Mollicutes* class are cell-wall-less organisms, therefore they are intrinsically resistant to β-lactam antibiotics. Generally, *Mycoplasma* species are sensitive to protein and nucleic acid synthesis inhibiting antibiotics, like pleuromutilins, tetracyclines, fluoroquinolones, aminoglycosides, phenicols, macrolides and lincosamides^9^. In the past years, antibiotic susceptibility testing of several *M. hyosynoviae* isolates was performed. Isolates from Asia, Europe and the United States collected between the 1960s and the 2020s have been examined and generally low minimal inhibitory concentrations (MICs) of the tested antibiotics were detected^10–22^.

The aim of this study was to determine the susceptibility of recent isolates of *M. hyosynoviae* from five European countries to ten antibiotics licensed for veterinary use in swine arthritis, and to compare the results with literature data.

## Results

**Fig. 1:**
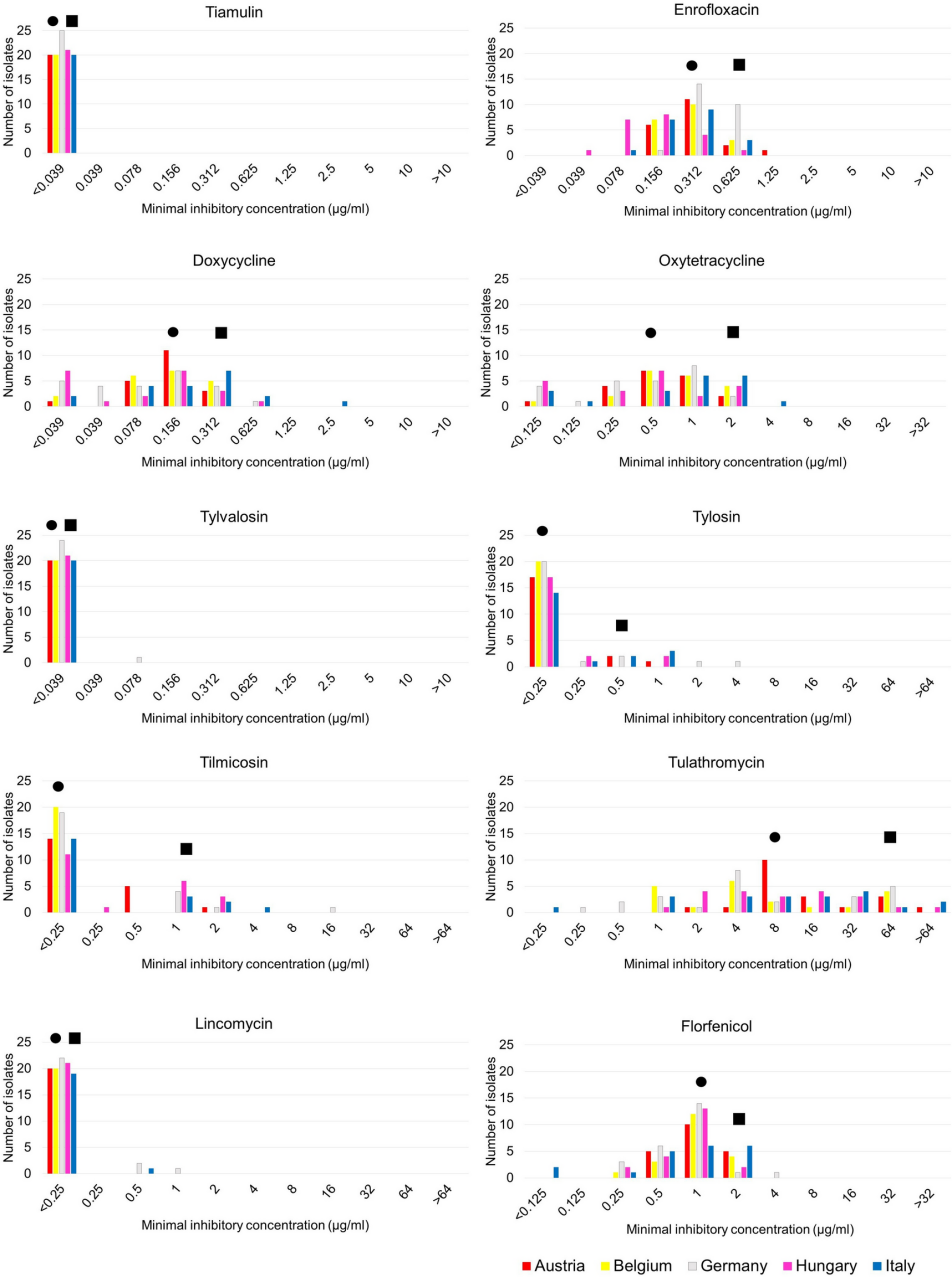
Minimal inhibitory concentration (MIC) distribution of the 106 tested *Mycoplasma hyosynoviae* isolates for tiamulin, enrofloxacin, doxycycline, oxytetracycline, tylvalosin, tylosin, tilmicosin, tulathromycin, lincomycin and florfenicol by country of origin. Circles indicate the MIC_50_ values and squares the MIC_90_ values of all tested isolates.

The determined MIC parameters (MIC range, MIC_50_ and MIC_90_) are listed separately by individual country (Table 1). Detailed MIC results of the tested antibiotic agents are described in Supplementary table 1. Regardless of the country of origin, similar susceptibility distribution patterns were determined, and most antibiotics showed monomodal MIC patterns that shifted to the left, except for tulathromycin where a uniform MIC pattern was observed (Fig. 1). The analysed *M. hyosynoviae* strains were highly susceptible to tiamulin (MIC_90_ ≤ 0.039 µg/ml), tylvalosin (MIC_90_ ≤ 0.039 µg/ml) and lincomycin (MIC_90_ ≤ 0.25 µg/ml). The isolates were also susceptible to tylosin (MIC_90_ 0.5 µg/ml) and tilmicosin (MIC_90_ 1 µg/ml). Moderate MIC values were detected for doxycycline (MIC_90_ 0.312 µg/ml), oxytetracycline (MIC_90_ 2 µg/ml), enrofloxacin (MIC_90_ 0.625 µg/ml) and florfenicol (MIC_90_ 2 µg/ml). Concerning tulathromycin, only high concentrations of the antibiotic were able to inhibit the growth of the tested isolates (MIC

**Table 1:** Minimal inhibitory concentration (MIC; µg/ml) values of ten antimicrobial agents against 106 *Mycoplasma hyosynoviae* isolates and the type strain (NCTC 10,167). Total values and values for each country are given.

Country of origin	MIC parameter	Tia	Enr	Dox	Oxy	Tyv	Tyl	Til	Tul	Lin	Flo
Type strain	MIC Range	≤ 0.039	0.312–0.625	0.039–0.156	0.125–1	≤ 0.039	≤ 0.25	≤ 0.25	2–32	≤ 0.25	0.25–1
Austria (20 isolates)	MIC Range	≤ 0.039	0.156–1.25	≤ 0.039–0.312	≤ 0.125–2	≤ 0.039	≤ 0.25–1	≤ 0.25–2	2- > 64	≤ 0.25	0.5–2
	MIC_50_	≤ 0.039	0.312	0.156	0.5	≤ 0.039	≤ 0.25	≤ 0.25	8	≤ 0.25	1
	MIC_90_	≤ 0.039	0.625	0.312	1	≤ 0.039	0.5	0.5	64	≤ 0.25	2
Belgium (20 isolates)	MIC Range	≤ 0.039	0.156–0.625	≤ 0.039–0.312	≤ 0.125–2	≤ 0.039	≤ 0.25	≤ 0.25	1–64	≤ 0.25	0.25–2
	MIC_50_	≤ 0.039	0.312	0.156	0.5	≤ 0.039	≤ 0.25	≤ 0.25	4	≤ 0.25	1
	MIC_90_	≤ 0.039	0.625	0.312	2	≤ 0.039	≤ 0.25	≤ 0.25	64	≤ 0.25	2
Germany (25 isolates)	MIC Range	≤ 0.039	0.156–0.625	≤ 0.039–0.625	≤ 0.125–2	≤ 0.039–0.078	≤ 0.25–4	≤ 0.25–16	0.25–64	≤ 0.25–1	0.25–4
	MIC_50_	≤ 0.039	0.312	0.078	0.5	≤ 0.039	≤ 0.25	≤ 0.25	4	≤ 0.25	1
	MIC_90_	≤ 0.039	0.625	0.312	1	≤ 0.039	0.5	1	64	0.5	1
Hungary (21 isolates)	MIC Range	≤ 0.039	0.039–0.625	≤ 0.039–0.625	≤ 0.125–2	≤ 0.039	≤ 0.25–1	≤ 0.25–2	1- > 64	≤ 0.25	0.25–2
	MIC_50_	≤ 0.039	0.156	0.156	0.5	≤ 0.039	≤ 0.25	≤ 0.25	8	≤ 0.25	1
	MIC_90_	≤ 0.039	0.312	0.312	2	≤ 0.039	0.25	2	32	≤ 0.25	2
Italy (20 isolates)	MIC Range	≤ 0.039	0.078–0.625	≤ 0.039–2.5	≤ 0.125–4	≤ 0.039	≤ 0.25–1	≤ 0.25–4	≤ 0.25- > 64	≤ 0.25–0.5	≤ 0.125–2
	MIC_50_	≤ 0.039	0.312	0.156	1	≤ 0.039	≤ 0.25	≤ 0.25	8	≤ 0.25	1
	MIC_90_	≤ 0.039	0.625	0.625	2	≤ 0.039	1	2	64	≤ 0.25	2
All 106 isolates	MIC Range	≤ 0.039	0.039–1.25	≤ 0.039–2.5	≤ 0.125–4	≤ 0.039–0.078	≤ 0.25–4	≤ 0.25–16	≤ 0.25–64	≤ 0.25–1	≤ 0.125–4
	MIC_50_	≤ 0.039	0.312	0.156	0.5	≤ 0.039	≤ 0.25	≤ 0.25	8	≤ 0.25	1
	MIC_90_	≤ 0.039	0.625	0.312	2	≤ 0.039	0.5	1	64	≤ 0.25	2

Tia: tiamulin, Enr: enrofloxacin, Dox: doxycycline, Oxy: oxytetracycline, Tyv: tylvalosin, Tyl: tylosin, Til: tilmicosin, Tul: tulathromycin, Lin: lincomycin, Flo: florfenicol.

_90_ 64 µg/ml).

**Fig. 2:**
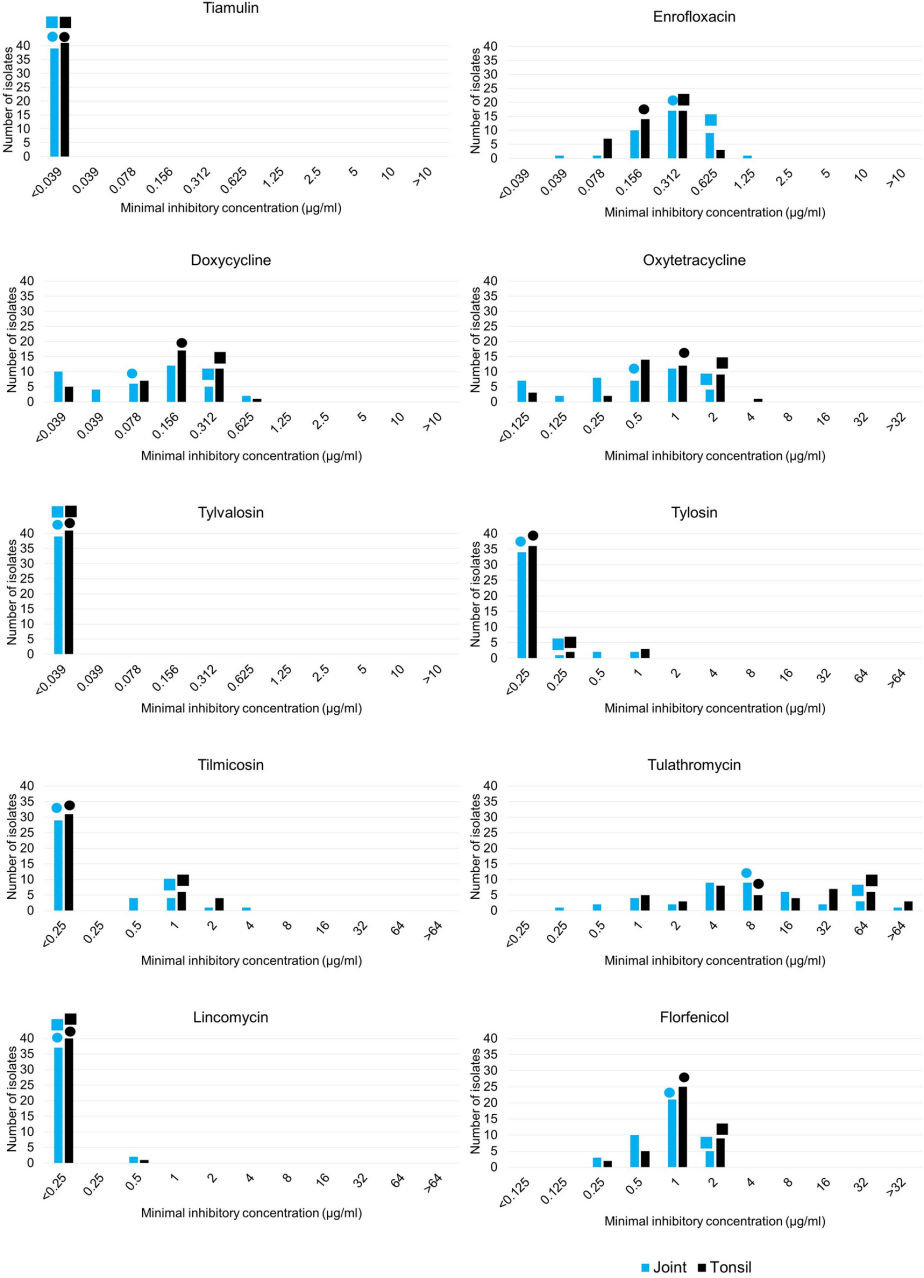
Minimal inhibitory concentration (MIC) distribution of the 80 *Mycoplasma hyosynoviae* isolates for tiamulin, enrofloxacin, doxycycline, oxytetracycline, tylvalosin, tylosin, tilmicosin, tulathromycin, lincomycin and florfenicol by organ of origin (joint or tonsil). Circles indicate the MIC_50_ values and squares the MIC_90_ values of the isolates.

A comparison of MIC values determined for isolates collected from joints (n = 39) and tonsils (n = 41) was performed (Fig. 2). MIC_90_ values only differed in the case of enrofloxacin, where the MIC_90_ value of the tonsil samples was 0.312 µg/ml, while for the joint samples it was 0.625 µg/ml. MIC_50_ values differed in the case of enrofloxacin (joint: 0.312 µg/ml; tonsil: 0.156 µg/ml), doxycycline (joint: 0.078 µg/ml; tonsil: 0.156 µg/ml) and oxytetracycline (joint: 0.5 µg/ml; tonsil: 1 µg/ml; Fig. 2).

For enrofloxacin, the distribution of MIC values was statistically different among countries (Table 2). In particular, the Hungarian isolates displayed low MIC values with higher frequencies compared to all other countries (Austria, Belgium, Germany, and Italy). On the contrary, German isolates proved to be the least sensitive to enrofloxacin (Table 3).

**Table 2:** Extended Cochran-Armitage test of the frequency of different classes of the antimicrobials’ minimal inhibitory concentration by country.

Antibiotic	X^2^	*p*-value	Adjusted *p*-value
**Enrofloxacin**	**32.42**	** < 0.01**	** < 0.01**
Doxycycline	8.91	0.06	0.19
Oxytetracycline	5.75	0.22	0.34
Tylvalosin	3.27	0.51	0.56
Tylosin	5.66	0.23	0.34
Tilmicosin	10.86	0.03	0.13
Tulathromycin	4.25	0.37	0.48
Lincomycin	7.05	0.13	0.30
Florfenicol	3.01	0.56	0.56

Significant differences are highlighted in bold lettering.

**Table 3:** Pairwise estimate comparisons of the proportional odds model relating the frequency of observation of the different classes of enrofloxacin minimal inhibitory concentrations to the variable country.

Contrasts	Estimate	Standard Error	Z-value	Adjusted *p*-value
Austria–Belgium	0.18	0.60	0.29	0.77
**Austria–Germany**	**− 0.33**	**0.58**	**− 2.29**	**0.03**
**Austria–Hungary**	**2.65**	**0.67**	**3.98**	** < 0.01**
Austria–Italy	0.39	0.61	0.65	0.65
**Belgium–Germany**	**− 1.50**	**0.58**	**− 2.60**	**0.02**
**Belgium–Hungary**	**2.47**	**0.66**	**3.75**	** < 0.01**
Belgium–Italy	0.22	0.61	0.36	0.77
**Germany–Hungary**	**3.97**	**0.69**	**5.74**	** < 0.01**
**Germany–Italy**	**1.72**	**0.59**	**2.92**	** < 0.01**
**Hungary–Italy**	**− 2.25**	**0.66**	**− 3.42**	** < 0.01**

Significant differences are highlighted in bold lettering.

Based on the multi-locus sequence typing (MLST) all isolates represented unique sequence types (ST), except for Bl 5 and Bl 14 which were both typed as ST 75. The twenty isolates typed in this study represented six new allele types on *dnaA* gene, five on *ftsY* gene, one on *fusA* gene, one on *gyrB* gene, six on *rpoB* gene and two on *uvrA* gene. In addition, these isolates represented 19 new STs. No correlation was found between ST and country of origin or ST and sample of origin. The two Belgian isolates with the same ST (ST 75) showed similar susceptibility to the tested antibiotics, except in the case of tulathromycin (Bl 5: 64 µg/ml, Bl 14: 1 µg/ml). Detailed results of the MLST can be found in Supplementary table 3 and Supplementary Fig. 1. All new allele types and STs were submitted to PubMLST.

## Discussion

In vitro susceptibility testing of veterinary mycoplasmas requires special expertise, is time consuming and still not standardised. Therefore, continuous and comparable antibiotic susceptibility testing of isolates is essential not just for the selection of antibiotics for therapeutic use, but also for the establishment of clinical breakpoint values in the future. Currently there are no official breakpoint values available for veterinary mycoplasmas. Based on the CVMP Guideline to demonstrate efficacy of veterinary medical products (CVMP/627/01-Rev1 ^23^), MIC data of antibiotics is essential for treatment justification. This study revealed the antibiotic susceptibility profiles of 106 recent *M. hyosynoviae* isolates from five European countries with considerable pig production sectors. A fraction of the isolates was typed by MLST, and showed high diversity (Supplementary Fig. 1) similarly to previous findings^24^.

Reports on the antibiotic susceptibility of *M. hyosynoviae* have been published since the 1960s in Asia (Japan, Thailand), Europe (mainly from Denmark and occasionally from Germany, Italy, Spain and Portugal) and the United States^10–22^ are available. The evaluated isolates were susceptible to most of the tested antibiotics with few exceptions. Elevated MICs were determined against isolates from Thailand and the United States, the fourth and the third largest consumer of veterinary antibiotics in the world in 2017, respectively^25^. Isolates collected between 1967 and 1970 in the United States showed decreased susceptibility to oxytetracycline^10^. Contrastingly, isolates collected between 1997 and 2011 in the United States showed decreased susceptibility to tilmicosin and tulathromycin^19^. More recently, isolates collected between 2008 and 2020 in Thailand showed decreased susceptibility to doxycycline, oxytetracycline, florfenicol, lincomycin, tilmicosin and tylosin^21,22^.

To compare susceptibility results through the years, publications which used the same antibiotic susceptibility testing method (broth micro-dilution based on the guidelines of Hannan^26^) and determined MIC values of the *M. hyosynoviae* type strain (NCTC 10,167 = S16) in the same range as the present study (Table 4) were included^11,13,15,16^. Among the comparable antibiotics (tiamulin, oxytetracycline, enrofloxacin, tylosin, tilmicosin, tylvalosin and lincomycin) the detected MICs were generally low over the reviewed 55-year period. The only exception was oxytetracycline where MIC_90_ values were moderate among the isolates before 1997 (MIC_90_ 5 µg/ml), similar to the value detected in this study (MIC_90_ 2 µg/ml). A slight increase, about one-fold dilution, in the MIC_90_’s of enrofloxacin was detected during the comparison of the isolates from before 1997 (MIC_90_ 0.25 µg/ml) and in our study (2018–2023; MIC_90_ 0.625 µg/ml). The constantly low MIC values of tiamulin and lincomycin over the last 55 years, and tylvalosin over the last ten years indicate high susceptibility in vitro. However, to predict therapeutic effectiveness the MIC data needs to be put into relationship with pharmacokinetic data. Pharmacokinetic properties of florfenicol^27,28^, tiamulin^29,30^ and tylvalosin^31^ in porcine synovial fluid have been determined. Based on these investigations, florfenicol and tiamulin can reach therapeutic concentrations in the synovial fluid at treatment dosage^27–29^, while tylvalosin did not reach therapeutic concentration after oral administration at the treatment dosage for 5 days^31^. Field studies on *M. hyosynoviae* infection have been documented only for tiamulin, indicating its effectiveness against this pathogen^32^.

**Table 4:** Comparison of minimal inhibitory concentrations (MIC; µg/ml) of seven antimicrobial agents from previous publications and the present study. MIC values against *Mycoplasma hyosynoviae* type strain (NCTC 10,167 = S16) gained in the different studies are also given.

Antibiotic		Tia	Oxy	Enr	Tyl	Til	Tyv	Lin
Denmark, 1968–1971 (n = 21)^16^	MIC_50_	0.0078	ND	0.25	0.0625	ND	ND	0.25
	MIC_90_	0.0156	ND	0.25	0.125	ND	ND	0.25
	Type strain	0.0078	ND	0.125	0.0625	ND	ND	0.25
Denmark, before 1994 (n = 6) ^11^	MIC_50_	0.0025	ND	0.25	0.025	ND	ND	0.05
	MIC_90_	0.0050	ND	0.50	0.50	ND	ND	0.10
	Type strain	0.0025	ND	0.25	0.025	ND	ND	0.10
Denmark, 1995–1996 (n = 21) ^16^	MIC_50_	0.0078	ND	0.25	0.5	ND	ND	0.25
	MIC_90_	0.0156	ND	0.25	1.0	ND	ND	0.50
	Type strain	0.0078	ND	0.125	0.0625	ND	ND	0.25
Denmark, France, Japan, Germany, USA, before 1997 (n = 18)^13^	MIC_50_	0.005	0.50	0.10	0.25	ND	ND	ND
	MIC_90_	0.025	5	0.25	1	ND	ND	ND
	Type strain	0.025	0.5	0.25	0.05	ND	ND	ND
Italy, Portugal, Spain, 2013–2018 (n = 40) ^15^	MIC_50_	0.125	ND	ND	0.5	1	0.016	0.5
	MIC_90_	0.5	ND	ND	1	2	0.06	1
	Type strain	0.03	ND	ND	0.25	0.25	0.0016	0.125
Austria, Belgium, Germany, Hungary, Italy, 2018–2023 (n = 106)^a^	MIC_50_	≤ 0.039	0.5	0.312	≤ 0.25	≤ 0.25	≤ 0.039	≤ 0.25
	MIC_90_	≤ 0.039	2	0.625	0.5	1	≤ 0.039	≤ 0.25
	Type strain	≤ 0.039	0.5	0.312	≤ 0.25	≤ 0.25	≤ 0.039	≤ 0.25

^a^present study;

Tia: tiamulin, Oxy: oxytetracycline, Enr: enrofloxacin, Tyl: tylosin, Til: tilmicosin, Tyv: tylvalosin, Lin: lincomycin; ND: not determined.

In the present study a comparison between the MICs of *M. hyosynoviae* isolates from tonsils and joints was performed, presuming that the isolates from tonsils mostly represent asymptomatic cases (commensal isolates), while the isolates from joint samples originated from diseased animals (clinical isolates). The comparison revealed no greater than one dilution differences in the MIC_90_ and MIC_50_ values between the commensal and clinical isolates, which suggests that antibiotic resistance may not be an important factor for invasiveness of *M. hyosynoviae*. Likewise, clinical *Escherichia (E.) coli* isolates from dog pyometra cases had similar antibiotic susceptibility profiles versus isolates from faecal matter of healthy bitches^33^.

In a previous paper Schultz and co-workers noted that in the case of some antibiotics, duplicate testing of the clinical isolates and the type strain may show higher than one-fold dilution differences. These differences were noticed in the MICs of florfenicol, tilmicosin, oxytetracycline (one isolate for each antibiotic from 23 tested), neomycin and chlortetracycline (2/23 isolates), and most frequently in the case of tulathromycin (4/23 isolates and the type strain) ^19^. In the present study the MIC values against duplicates of isolates did not differ from each other in higher than one dilution step; however, the MIC values against the *M. hyosynoviae* type strain showed differences higher than one dilution step in the case of florfenicol (a two-fold dilution difference) and tulathromycin (two two-fold dilution differences; Supplementary table 2). This is the second time of publishing antibiotic susceptibility data for tulathromycin, and the repeated testing of the *M. hyosynoviae* type strain revealed the same inconsistencies that were found previously. This underlines the theory from Schultz and co-workers that in vitro testing of tulathromycin by the broth micro-dilution method against this particular species might provide unreliable results.

The detected differences between the susceptibility of the *M. hyosynoviae* isolates from distinct geographical origins in the current study are probably influenced by the characteristics of antibiotic usage in the examined countries. In Germany, from where the isolates showed the statistically highest MICs of enrofloxacin, the treatment frequency with enrofloxacin increased between 2013 and 2015, when fluoroquinolones were the 9^th^ most used antibiotic group from the 12 monitored drug classes in fatteners. According to this survey enrofloxacin was the most commonly used fluoroquinolone in Germany^34^. In recent years the use of enrofloxacin has been restricted and the number of sales dropped by 24% by 2020. Nevertheless, the probability of *E. coli* isolates with MIC values higher than 0.25 µg/ml (non-wild-type according to EUCAST ECOFF) significantly increased when data from 2016 was compared with data from 2020^35^. This may indicate that the higher MIC values in the German *M. hyosynoviae* isolates for enrofloxacin are due to the more frequent use of this antibiotic in the past. Interestingly, more flouroquinolones were sold for veterinary use in Hungary (the country with the statistically lowest MIC values for enrofloxacin) than in Germany in each year since 2013 (ESVAC database: https://esvacbi.ema.europa.eu/analytics/saw.dll?Dashboard). However, in this database there is no information about the application of the sold antibiotics, and there are no available reports from Hungary to compare the use frequency of enrofloxacin in the swine industry and the MIC data of the isolates.

Similarly to *M. hyosynoviae*, *M. hyorhinis* can colonise the tonsils and upper respiratory tract of swine, and cause arthritis in weaners^3^. There are differences in the colonisation pattern of both mycoplasmas. *M. hyorhinis* colonises the upper respiratory tract and tonsils of piglets around three to 4 weeks-of-age^36–39^, while *M. hyosynoviae* colonises the tonsils of piglets older than 6 weeks-of-age^38–41^. After colonisation both bacteria persist in the upper respiratory tract and tonsils of the animals throughout their life^38,39^. The other difference between the two mycoplasmas is their metabolic activity, while *M. hyorhinis* ferments glucose, *M. hyosynoviae* hydrolyses arginine^2^.

The antibiotic susceptibility of *M. hyorhinis* isolates from the same European countries (except Austria and Poland) was published recently^42^. The MIC_90_ values of those *M. hyorhinis* isolates were similar to the *M. hyosynoviae* isolates examined here for florfenicol. On the other hand, MIC_90_ values for *M. hyorhinis* isolates were lower for tetracyclines, two dilution steps lower for doxycycline and three dilution steps lower for oxytetracycline. For the other tested antibiotics, the MIC_90_ values for *M. hyorhinis* were higher than MIC_90_ values for *M. hyosynoviae* determined in this study. In case of tiamulin, three-fold higher MIC_90_ value was observed, for enrofloxacin the *M. hyorhinis* isolates showed one-fold higher MIC_90_ value. In case of the tested macrolides (except tulathromycin) and lincomycin the *M. hyorhinis* isolates showed very high MIC_90_ values (MIC_90_ > 64 µg/ml for tylosin, tilmicosin and lincomycin and MIC_90_ 5 µg/ml for tylvalosin), while the *M. hyosynoviae* isolates showed low MIC_90_ values (MIC_90_ 0.5 µg/ml for tylosin, MIC_90_ 1 µg/ml for tilmicosin, MIC_90_ ≤ 0.039 µg/ml for tylvalosin and MIC_90_ ≤ 0.25 µg/ml for lincomycin)^42^. MIC_90_ values for tulathromycin were not compared because of the above mentioned inconsistencies for *M. hyosynoviae*. The differences between the antibiotic susceptibility of *M. hyosynoviae* and *M. hyorhinis* might be explained by differences in the colonisation time, predisposing species-specific characteristics, or metabolism.

In conclusion, based on the 106 tested European *M. hyosynoviae* isolates, lincomycin, tiamulin, tylvalosin, tylosin and tilmicosin proved to be the most effective antibiotic compounds in vitro. The detected uniform MIC values for tulathromycin and the country specific differences for enrofloxacin emphasise the importance of susceptibility monitoring on a Pan-European level and highlight the need for the introduction of standardised MIC testing and the development of veterinary-specific breakpoints for MIC result interpretation.

## Methods

### Collection of specimens

In total 106 M*. hyosynoviae* isolates collected between 2018 and 2023 were examined. The isolates originated from pigs on farms in Austria (n = 20), Belgium (n = 20), Germany (n = 25), Hungary (n = 21) and Italy (n = 20). The isolates were collected from different body sites: blood (n = 4), bronchoalveolar lavage fluid (n = 2), joint (n = 39), lung (n = 8), nasal cavity (n = 7), pericardium (n = 1), tonsil (n = 41) and in the case of five samples, the origin was not specified. Summary of number of farms and number of specimen type per country was summarised in Table 5 and detailed in Supplementary table 1.

**Table 5:** Summary of number of farms (provinces in case of Italy) and specimen types per each country.

	Number of farms	Number of specimen types
Austria	19	2 (a,b)
Belgium	no data	1 (c)
Germany	24	4 (a,d,e,f)
Hungary	19	3 (a, c, e)
Italy	10	5 (a, c, e, g, h)

a-joint, b-nasal cavity, c-tonsil, d-blood, e-lung, f-not specified, g-broncho-alveolar lavage, h-pericardium.

Samples were collected from both clinically affected animals and apparently healthy ones. Ethical approval and specific permission were not required for the study in accordance with the decision of the Animal Care and Use Committee of the Veterinary Medical Research Institute, Hungary, as all affected samples, used for the isolation, were collected by the authors with the consent of the owners during routine diagnostic examinations of carcasses.

### Cultivation of *Mycoplasma hyosynoviae*

In case of the Austrian, German and Italian samples the isolation was carried out in the partner laboratories according to their routine protocol. From Belgium, the tonsils were shipped to the central laboratory in Hungary, and the isolation was carried out there. The main differences between the isolation protocols were the used media and the incubation times. In Austria the samples were placed into 2SP medium and passaged into SP4-Z medium and agar with 1% arginine^43^. In Germany and Italy Mycoplasma Experience media (Mycoplasma Experience Ltd., Bletchingley, UK) were used. According to the Italian protocol this broth was supplemented with 8% L-arginine and 0.8% Bacto mucin. In Hungary MolliScience General Mycoplasma (GM) Liquid and Solid Media (MolliScience Kft. Biatorbágy, Hungary) were used. In each case the incubation was carried out at 37 °C with 5% CO_2_ for the agar plates, for three to four weeks. Species identification was based on PCR in Germany and Hungary^44^, PCR and denaturing gradient gel electrophoresis in Italy and Matrix-assisted laser desororption ionization time-of-flight mass spectrometry in Austria.

### Shipment of *Mycoplasma hyosynoviae* strains

Isolates were transferred to the central laboratory for antimicrobial susceptibility testing. Before shipment fresh passages were made and the cultures were shipped at -20 °C. Upon arrival the viability and purity of the isolates were checked before the MIC testing. The presence of other *Mycoplasma* species in the cultures was excluded by a set of polymerase chain reactions (PCR) preceded by DNA extraction. DNA extraction was performed by Chelex Resin (Chelex 100 Chelating Resin, molecular biology grade, Bio-Rad Hungary Ltd., Budapest, Hungary) according to the manufacturers’ instructions. All isolates were identified by *M. hyosynoviae* specific real-time PCR^44^. The presence of other swine mycoplasma species was excluded by real-time PCR specific for *M. hyopneumoniae*^45^, *M. hyorhinis*^46^, and by conventional PCR specific for *M. flocculare*^47^ and *M. hyopharyngis*^48^. To detect the presence of other possible contaminant *Mycoplasma* species, a universal *Mycoplasma* spp. specific PCR was performed targeting the 16S-23S intergenic spacer region^49^.

### Determination of minimal inhibitory concentrations

Throughout the tests, MolliScience General Mycoplasma (GM) Liquid Media (MolliScience Kft.) was used as culture medium. The number of colour changing units (CCU) was calculated by plate micro-dilution from the highest dilution showing colour change (red to pink shift)^26^.

The following antimicrobials were tested: one pleuromutilin (tiamulin), two tetracyclines (doxycycline, oxytetracycline), one phenicol (florfenicol), one fluoroquinolone (enrofloxacin), four macrolides (tylosin, tilmicosin, tylvalosin, tulathromycin), and one lincosamide (lincomycin). Tylvalosin originated from ECO® Animal Health Ltd. (London, UK) tulathromycin originated from Pfizer® Inc. (New York, NY, USA) and the rest of the compounds originated from Vetranal®, Sigma-Aldrich (St. Louis, MO, USA). Stock solutions at a concentration of 1 mg/ml were prepared, aliquoted and stored frozen at -70 °C until use. Twofold dilutions were freshly prepared before the tests in the range of 0.039–10 µg/ml for tiamulin, tylvalosin, doxycycline and enrofloxacin, 0.125–32 µg/ml for oxytetracycline and florfenicol and 0.25–64 µg/ml for tylosin, tilmicosin, tulathromycin and lincomycin. The broth micro-dilution test was performed in a 96-well microtiter plate containing a twofold dilution series of the antibiotic, with sterility, pH and growth control. The clinical isolates were tested in duplicates, and *M. hyosynoviae* type strain (NCTC 10,167) was included in the tests as quality and reproducibility control. All isolates were tested at the viable count of 10^5^ CCU/ml. The MIC value of each isolate was defined as the lowest concentration of the antibiotic where no colour change (no growth) was recorded by the time the growth control changed colour^26^. When a one-fold dilution difference was detected between the duplicates, the MIC value was determined based on the growth. When more than a one-fold dilution difference was detected between the duplicates, the test was repeated. MIC_50_ and MIC_90_ values were defined as the lowest concentrations that inhibited the growth of 50% and 90% of the tested isolates, respectively^26^.

As there are no available official breakpoints for veterinary mycoplasmas, a categorisation based on the MIC_50_/MIC_90_ values was made for easier description of the differences in susceptibility. The categories were as follows:

- Isolates with MIC_90_ values below the lowest antibiotic concentration tested were considered highly susceptible;
- Isolates with MIC_90_ values in the first third of the dilution series and MIC_50_ values below the lowest antibiotic concentration tested were considered susceptible, and the MIC_90_ values were categorised as low;
- Isolates with MIC_90_ values in the middle of the dilution range considered moderate and MIC_50_ values in the upper third of the dilution series were considered high.

### Data analysis

The statistical analyses presented in this work were conducted under R environment^50^. First, an extended Cochran Armitage test was performed with a null hypothesis proposing that the distribution of the frequency of each MIC value was independent of the country (package “coin”)^51^. This test was performed for all antibiotics except tiamulin, whose MIC values were the same for all tested isolates. Since multiple antibiotics were considered at the same time, p-values were adjusted for multiple comparisons by the Benjamini–Hochberg method. In cases where significant differences were found, a proportional odds model for each antibiotic was constructed relating the cumulative frequency distribution of each MIC value to the variable country. The models were constructed with the function *clm* of the package “ordinal”^52^, implemented a logit link function and assumed equidistant thresholds (for model parameter estimation, see Supplementary table 4). The validity of the proportional odds assumption was assessed with the function *nominal* of the same package. Pairwise estimate comparison among countries was carried out with the package “emmeans”^53^. Estimates are presented in the logit form.

### Molecular investigation

Multi-locus sequence typing (MLST) was performed based on the previously published scheme^24^. Five isolates were chosen from each country based on isolation year and sample of origin. The MLST profiles of the Austrian isolates were determined earlier and deposited in PubMLST (https://pubmlst.org/organisms/mycoplasma-hyosynoviae). For the rest of the isolates first the whole genome sequences were determined. DNA was extracted from the samples with the QIAmp DNA Mini Kit (Qiagen Inc., Valencia, CA, USA). Short read sequencing was implemented on an Illumina NextSeq 500 platform (Illumina Inc., San Diego, CA, USA). Library was prepared with the Nextera XT DNA Library Preparation Kit and the Nextera XT Index Kit v2 Set A as described elsewhere^54^. To gain 2 × 150 bp paired-end reads, the library pool at a final concentration of 1.8 pM was loaded onto a NextSeq 500/550 High Output flow cell. The short reads were mapped to the genome of the *M. hyosynoviae* type strain (GenBank ID: GCA_024297005.1) in Geneious Prime software (version 2022.2.2)^55^. Reference sequences of each house-keeping gene sequence was obtained from the PubMLST database and aligned to the whole genome sequences in Geneious Prime software. The phylogenetic tree was constructed using the Maximum Likelihood method and Hasegawa-Kishino-Yano substitution model with bootstrapping (1000 replications) in Mega X software^56^. Phylogenetic analysis was performed with all available sequences in the PubMLST database and separately for the 25 isolates from this study (Supplementary Fig. 1).

## Supplementary Information

Supplementary Information 1.

Supplementary Information 2.

Supplementary Information 3.

Supplementary Information 4.

Supplementary Information 5.

Supplementary Information 6.

## Data Availability

All data generated or analysed during this study are included in this published article and its Supplementary information files. The MLST profiles of the tested isolates were deposited in PubMLST (https://pubmlst.org/organisms/mycoplasma-hyosynoviae).
