# Supplementary material for: Antimicrobial susceptibility profiles of Mycoplasma hyosynoviae strains isolated from five European countries between 2018 and 2023
Source: Sci Rep. 2025 Jan 7;15:1243. doi: 10.1038/s41598-024-85052-1 (PMC11707295; doi:10.1038/s41598-024-85052-1)
Supplement: Supplementary file 1 — Supplementary Information 1. [file 41598_2024_85052_MOESM1_ESM.pdf]

Supplementary information

Supplementary figure 1: Molecular typing of *Mycoplasma hyosynoviae* isolates by multi-locus sequence typing.

A. Phylogenetic tree of all isolates available in the PubMLST database. The isolates from the present study are highlighted pink. B. Phylogenetic tree of the 25 isolates from the present study.

Supplementary table 1: Background information of the tested *Mycoplasma hyosynoviae* isolates and results of the broth micro-dilution test.

Supplementary table 2: Minimal inhibitory concentrations of the *Mycoplasma hyosynoviae* type strain to the tested antibiotics in each repeat.

Supplementary table 3: Allele and sequence types of the *Mycoplasma hyosynoviae* isolates based on multi-locus sequence typing.

Supplementary table 4: Parameter estimates with relative standard error, Wald statistic (Z-value) and p-value of the proportional odds model relating to the frequency of observation of the different minimal inhibitory concentration values of enrofloxacin to the variable country.
