# Supplementary figures and images for: Antimicrobial susceptibility profiles of Mycoplasma hyosynoviae strains isolated from five European countries between 2018 and 2023
Source: Sci Rep. 2025 Jan 7;15:1243. doi: 10.1038/s41598-024-85052-1 (PMC11707295; doi:10.1038/s41598-024-85052-1)

A

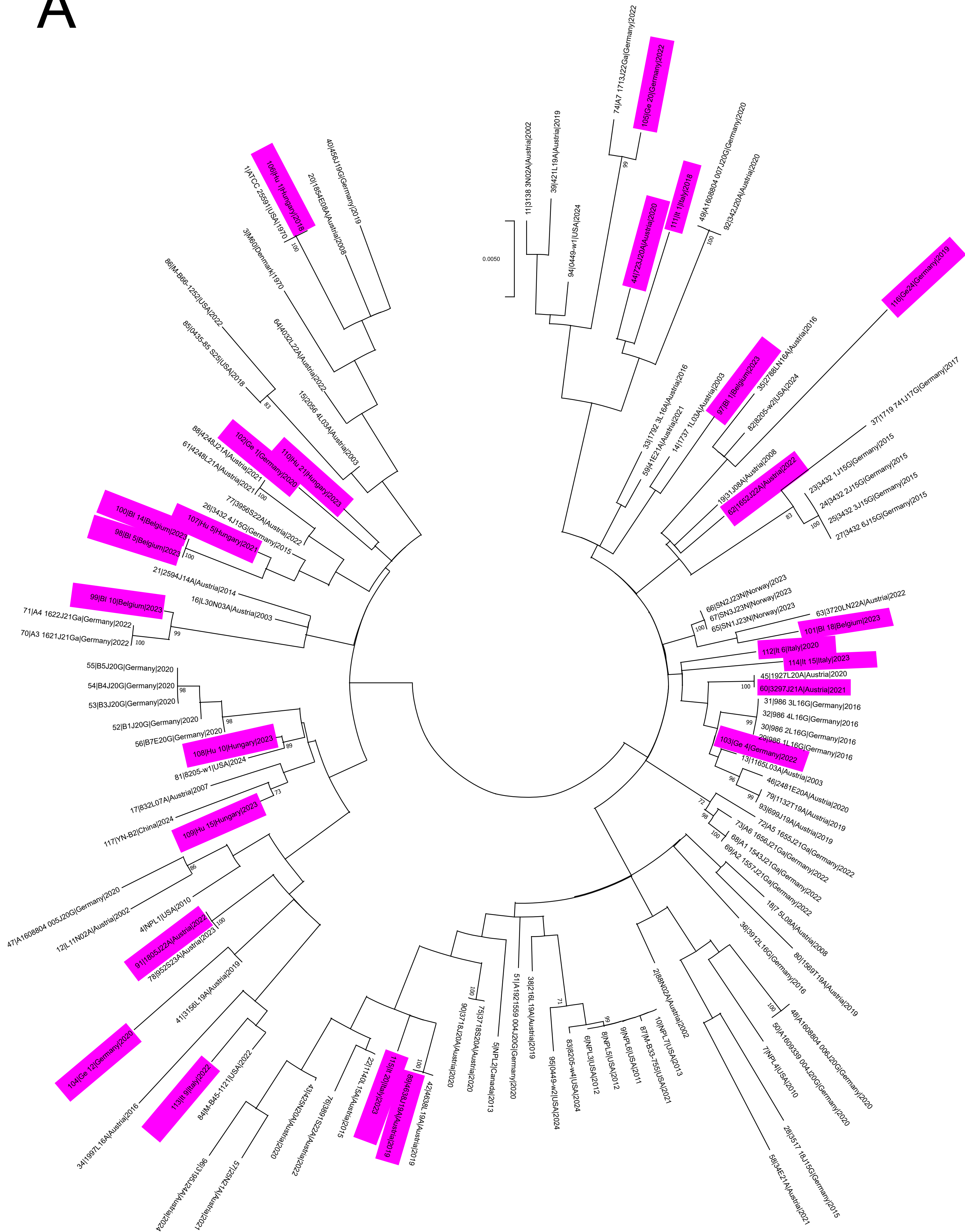

B

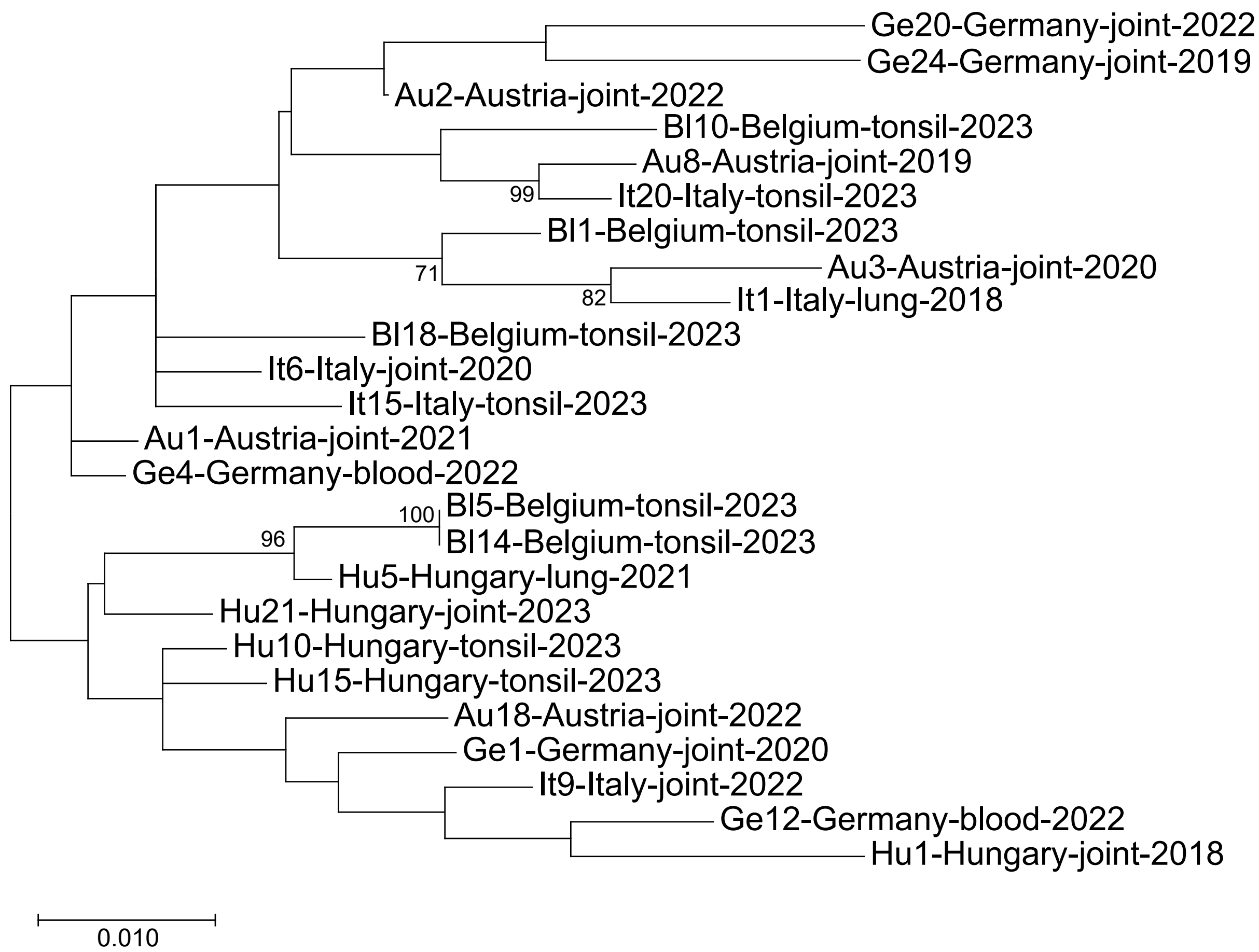

Supplement: Supplementary file 2 — Supplementary Information 2. [file 41598_2024_85052_MOESM2_ESM.pdf]
