# Supplementary material for: Antimicrobial susceptibility profiles of Mycoplasma hyosynoviae strains isolated from five European countries between 2018 and 2023
Source: Sci Rep. 2025 Jan 7;15:1243. doi: 10.1038/s41598-024-85052-1 (PMC11707295; doi:10.1038/s41598-024-85052-1)
Supplement: Supplementary file 3 — Supplementary Information 3. [file 41598_2024_85052_MOESM3_ESM.pdf]

**Supplementary table 1**

| ID | Country of origin | Sample type* | Isolation year | Farm/Province** | CCU/ml*** | Minimal Inhibitory Concentrations (µg/ml) | | | | | | | | | |
|---|---|---|---|---|---|---|---|---|---|---|---|---|---|---|---|
| | | | | | | Tiamulin | Tylosin | Tilmicosin | Tylvalosin | Tulathromyci | Lincomycin | Doxycycline | Oxytetracycline | Florfenicol | Enrofloxacin |
| Mycoplasma hyosynoviae reference strain | USA | joint | 1970 | | 10^7 | ≤0.039 | ≤0.25 | ≤0.25 | ≤0.039 | 8 | ≤0.25 | 0.078 | 0.5 | 0.25 | 0.312 |
| Au 1 | Austria | joint | 2021 | Ybbsitz | 10^8 | ≤0.039 | ≤0.25 | ≤0.25 | ≤0.039 | 16 | ≤0.25 | 0.156 | 0.5 | 0.5 | 1.25 |
| Au 2 | Austria | joint | 2022 | Weitersfeld | 10^9 | ≤0.039 | ≤0.25 | ≤0.25 | ≤0.039 | 32 | ≤0.25 | 0.156 | 1 | 1 | 0.156 |
| Au 3 | Austria | joint | 2020 | Thanstetten I | 10^9 | ≤0.039 | ≤0.25 | ≤0.25 | ≤0.039 | 64 | ≤0.25 | 0.156 | 1 | 1 | 0.312 |
| Au 4 | Austria | joint | 2021 | Sankt Martin an der Raab | 10^9 | ≤0.039 | ≤0.25 | 0.5 | ≤0.039 | 8 | ≤0.25 | 0.078 | 0.25 | 2 | 0.156 |
| Au 5 | Austria | joint | 2020 | Spielfeld | 10^8 | ≤0.039 | ≤0.25 | ≤0.25 | ≤0.039 | 8 | ≤0.25 | 0.078 | 0.25 | 2 | 0.156 |
| Au 6 | Austria | joint | 2020 | Pettenbach | 10^9 | ≤0.039 | 1 | 2 | ≤0.039 | >64 | ≤0.25 | 0.156 | 1 | 1 | 0.312 |
| Au 7 | Austria | joint | 2020 | Großrußbach | 10^6 | ≤0.039 | 0.5 | ≤0.25 | ≤0.039 | 8 | ≤0.25 | 0.156 | 0.5 | 1 | 0.625 |
| Au 8 | Austria | joint | 2019 | Leibnitz | 10^6 | ≤0.039 | ≤0.25 | ≤0.25 | ≤0.039 | 16 | ≤0.25 | 0.312 | 1 | 1 | 0.312 |
| Au 9 | Austria | joint | 2020 | Allhatsberg | 10^8 | ≤0.039 | ≤0.25 | 0.5 | ≤0.039 | 8 | ≤0.25 | 0.156 | 0.5 | 2 | 0.156 |
| Au 10 | Austria | nasal cavity | 2022 | Haag | 10^8 | ≤0.039 | ≤0.25 | ≤0.25 | ≤0.039 | 8 | ≤0.25 | 0.078 | 0.5 | 2 | 0.156 |
| Au 11 | Austria | joint | 2020 | Buchkirchen | 10^10 | ≤0.039 | ≤0.25 | 0.5 | ≤0.039 | 8 | ≤0.25 | 0.156 | 0.5 | 1 | 0.312 |
| Au 12 | Austria | joint | 2020 | Thanstetten II | 10^9 | ≤0.039 | ≤0.25 | 0.5 | ≤0.039 | 8 | ≤0.25 | 0.156 | 1 | 1 | 0.312 |
| Au 13 | Austria | joint | 2019 | Eberstalzell | 10^8 | ≤0.039 | ≤0.25 | ≤0.25 | ≤0.039 | 64 | ≤0.25 | 0.156 | 1 | 1 | 0.312 |
| Au 14 | Austria | nasal cavity | 2021 | Sankt Nikola an der Donau | 10^6 | ≤0.039 | 0.5 | 0.5 | ≤0.039 | 8 | ≤0.25 | 0.078 | 0.25 | 0.5 | 0.312 |
| Au 15 | Austria | nasal cavity | 2021 | Puch bei Weiz | 10^8 | ≤0.039 | ≤0.25 | ≤0.25 | ≤0.039 | 2 | ≤0.25 | 0.156 | 0.5 | 0.5 | 0.156 |
| Au 16 | Austria | nasal cavity | 2022 | Piberbach | 10^9 | ≤0.039 | ≤0.25 | ≤0.25 | ≤0.039 | 8 | ≤0.25 | 0.078 | 0.25 | 1 | 0.312 |
| Au 17 | Austria | nasal cavity | 2022 | kt Marienkirchen an der Pol | 10^8 | ≤0.039 | ≤0.25 | ≤0.25 | ≤0.039 | 8 | ≤0.25 | 0.312 | 2 | 2 | 0.312 |
| Au 18 | Austria | joint | 2022 | Wettmannstätten | 10^10 | ≤0.039 | ≤0.25 | ≤0.25 | ≤0.039 | 16 | ≤0.25 | 0.156 | 0.5 | 0.5 | 0.312 |
| Au 19 | Austria | nasal cavity | 2022 | Euratsfeld | 10^9 | ≤0.039 | ≤0.25 | ≤0.25 | ≤0.039 | 4 | ≤0.25 | ≤0.039 | ≤0.125 | 1 | 0.312 |
| Au 20 | Austria | nasal cavity | 2021 | Lannach | 10^8 | ≤0.039 | ≤0.25 | ≤0.25 | ≤0.039 | 64 | ≤0.25 | 0.312 | 2 | 0.5 | 0.625 |
| Bl 1 | Belgium | tonsil | 2023 | no data | 10^9 | ≤0.039 | ≤0.25 | ≤0.25 | ≤0.039 | 16 | ≤0.25 | 0.156 | 1 | 1 | 0.156 |
| Bl 2 | Belgium | tonsil | 2023 | no data | 10^9 | ≤0.039 | ≤0.25 | ≤0.25 | ≤0.039 | 4 | ≤0.25 | 0.156 | 1 | 1 | 0.156 |
| Bl 3 | Belgium | tonsil | 2023 | no data | 10^9 | ≤0.039 | ≤0.25 | ≤0.25 | ≤0.039 | 4 | ≤0.25 | 0.156 | 0.5 | 1 | 0.156 |
| Bl 4 | Belgium | tonsil | 2023 | no data | 10^9 | ≤0.039 | ≤0.25 | ≤0.25 | ≤0.039 | 4 | ≤0.25 | 0.078 | 0.5 | 1 | 0.156 |
| Bl 5 | Belgium | tonsil | 2023 | no data | 10^8 | ≤0.039 | ≤0.25 | ≤0.25 | ≤0.039 | 64 | ≤0.25 | 0.312 | 2 | 1 | 0.625 |
| Bl 6 | Belgium | tonsil | 2023 | no data | 10^9 | ≤0.039 | ≤0.25 | ≤0.25 | ≤0.039 | 4 | ≤0.25 | 0.156 | 1 | 1 | 0.156 |
| Bl 7 | Belgium | tonsil | 2023 | no data | 10^9 | ≤0.039 | ≤0.25 | ≤0.25 | ≤0.039 | 1 | ≤0.25 | ≤0.039 | 0.25 | 0.5 | 0.625 |
| Bl 8 | Belgium | tonsil | 2023 | no data | 10^9 | ≤0.039 | ≤0.25 | ≤0.25 | ≤0.039 | 4 | ≤0.25 | 0.078 | 0.25 | 1 | 0.156 |
| Bl 9 | Belgium | tonsil | 2023 | no data | 10^9 | ≤0.039 | ≤0.25 | ≤0.25 | ≤0.039 | 4 | ≤0.25 | 0.078 | 0.5 | 1 | 0.625 |
| Bl 10 | Belgium | tonsil | 2023 | no data | 10^8 | ≤0.039 | ≤0.25 | ≤0.25 | ≤0.039 | 8 | ≤0.25 | 0.156 | 1 | 0.5 | 0.312 |
| Bl 11 | Belgium | tonsil | 2023 | no data | 10^9 | ≤0.039 | ≤0.25 | ≤0.25 | ≤0.039 | 2 | ≤0.25 | 0.078 | 0.5 | 1 | 0.312 |
| Bl 12 | Belgium | tonsil | 2023 | no data | 10^8 | ≤0.039 | ≤0.25 | ≤0.25 | ≤0.039 | 8 | ≤0.25 | 0.078 | 0.5 | 0.5 | 0.312 |
| Bl 13 | Belgium | tonsil | 2023 | no data | 10^9 | ≤0.039 | ≤0.25 | ≤0.25 | ≤0.039 | 1 | ≤0.25 | 0.312 | 2 | 1 | 0.312 |
| Bl 14 | Belgium | tonsil | 2023 | no data | 10^9 | ≤0.039 | ≤0.25 | ≤0.25 | ≤0.039 | 1 | ≤0.25 | 0.312 | 2 | 1 | 0.312 |
| Bl 15 | Belgium | tonsil | 2023 | no data | 10^9 | ≤0.039 | ≤0.25 | ≤0.25 | ≤0.039 | 1 | ≤0.25 | 0.312 | 1 | 1 | 0.312 |
| Bl 16 | Belgium | tonsil | 2023 | no data | 10^10 | ≤0.039 | ≤0.25 | ≤0.25 | ≤0.039 | 1 | ≤0.25 | 0.078 | 0.5 | 0.25 | 0.312 |
| Bl 17 | Belgium | tonsil | 2023 | no data | 10^9 | ≤0.039 | ≤0.25 | ≤0.25 | ≤0.039 | 64 | ≤0.25 | 0.156 | 1 | 2 | 0.312 |
| Bl 18 | Belgium | tonsil | 2023 | no data | 10^9 | ≤0.039 | ≤0.25 | ≤0.25 | ≤0.039 | 64 | ≤0.25 | 0.156 | 0.5 | 2 | 0.312 |
| Bl 19 | Belgium | tonsil | 2023 | no data | 10^9 | ≤0.039 | ≤0.25 | ≤0.25 | ≤0.039 | 32 | ≤0.25 | 0.312 | 2 | 2 | 0.156 |
| Bl 20 | Belgium | tonsil | 2023 | no data | 10^9 | ≤0.039 | ≤0.25 | ≤0.25 | ≤0.039 | 64 | ≤0.25 | ≤0.039 | ≤0.125 | 2 | 0.312 |
| Ge 1 | Germany | joint | 2020 | Neustadt am Rübenberge | 10^9 | ≤0.039 | ≤0.25 | ≤0.25 | ≤0.039 | 32 | ≤0.25 | 0.312 | 1 | 1 | 0.312 |
| Ge 2 | Germany | blood | 2020 | Handrup | 10^9 | ≤0.039 | 0.5 | 1 | ≤0.039 | 64 | ≤0.25 | 0.312 | 1 | 1 | 0.312 |
| Ge 3 | Germany | joint | 2022 | Eimke | 10^9 | ≤0.039 | ≤0.25 | ≤0.25 | ≤0.039 | 64 | 0.5 | 0.312 | 2 | 1 | 0.625 |
| Ge 4 | Germany | blood | 2022 | Donauwörth | 10^10 | ≤0.039 | ≤0.25 | ≤0.25 | ≤0.039 | 32 | ≤0.25 | 0.156 | 1 | 1 | 0.312 |
| Ge 5 | Germany | not specified | 2020 | Borgholzhausen | 10^9 | ≤0.039 | ≤0.25 | ≤0.25 | ≤0.039 | 64 | ≤0.25 | 0.156 | 1 | 1 | 0.312 |
| Ge 6 | Germany | joint | 2020 | Lütjenholm | 10^9 | ≤0.039 | ≤0.25 | ≤0.25 | ≤0.039 | 4 | ≤0.25 | 0.156 | 0.25 | 1 | 0.312 |
| Ge 7 | Germany | joint | 2020 | Barnstorf | 10^10 | ≤0.039 | ≤0.25 | ≤0.25 | ≤0.039 | 0.5 | ≤0.25 | 0.625 | 2 | 0.5 | 0.312 |
| Ge 8 | Germany | joint | 2021 | Titting | 10^8 | ≤0.039 | ≤0.25 | ≤0.25 | ≤0.039 | 8 | ≤0.25 | 0.156 | ≤0.125 | 1 | 0.312 |
| Ge 9 | Germany | joint | 2023 | Cappeln | 10^9 | ≤0.039 | ≤0.25 | ≤0.25 | ≤0.039 | 4 | 0.5 | ≤0.039 | ≤0.125 | 1 | 0.625 |
| Ge 10 | Germany | joint | 2021 | Langwedel | 10^9 | ≤0.039 | ≤0.25 | ≤0.25 | ≤0.039 | 4 | ≤0.25 | 0.039 | 0.125 | 0.5 | 0.312 |
| Ge 11 | Germany | blood | 2022 | Mertingen | 10^8 | ≤0.039 | ≤0.25 | ≤0.25 | ≤0.039 | 64 | ≤0.25 | 0.312 | 1 | 0.5 | 0.312 |
| Ge 12 | Germany | blood | 2020 | Fürstenau | 10^9 | ≤0.039 | 2 | 2 | ≤0.039 | 64 | ≤0.25 | 0.078 | 0.25 | 0.25 | 0.625 |
| Ge 13 | Germany | joint | 2020 | Dötlingen | 10^9 | ≤0.039 | ≤0.25 | ≤0.25 | ≤0.039 | 2 | ≤0.25 | 0.039 | 1 | 0.25 | 0.312 |
| Ge 14 | Germany | not specified | 2021 | Esens | 10^9 | ≤0.039 | ≤0.25 | ≤0.25 | ≤0.039 | 32 | ≤0.25 | 0.156 | 0.5 | 0.25 | 0.312 |
| Ge 15 | Germany | joint | 2020 | Warendorf I | 10^8 | ≤0.039 | 0.5 | 1 | ≤0.039 | 0.25 | ≤0.25 | ≤0.039 | ≤0.125 | 1 | 0.625 |
| Ge 16 | Germany | joint | 2020 | Warendorf II | 10^10 | ≤0.039 | ≤0.25 | ≤0.25 | ≤0.039 | 1 | ≤0.25 | ≤0.039 | 0.25 | 1 | 0.625 |
| Ge 17 | Germany | joint | 2022 | Steinfeld | 10^8 | ≤0.039 | ≤0.25 | 1 | ≤0.039 | 4 | ≤0.25 | 0.039 | 0.5 | 1 | 0.625 |
| Ge 18 | Germany | joint | 2022 | Glandorf | 10^9 | ≤0.039 | 0.25 | 1 | ≤0.039 | 4 | ≤0.25 | 0.078 | 0.25 | 0.5 | 0.156 |
| Ge 19 | Germany | joint | 2022 | Weißenstadt | 10^9 | ≤0.039 | ≤0.25 | ≤0.25 | ≤0.039 | 4 | ≤0.25 | ≤0.039 | 1 | 1 | 0.312 |
| Ge 20 | Germany | joint | 2022 | Beckum | 10^8 | ≤0.039 | ≤0.25 | ≤0.25 | ≤0.039 | 0.5 | ≤0.25 | ≤0.039 | ≤0.125 | 1 | 0.625 |
| Ge 21 | Germany | not specified | 2019 | Bissendorf | 10^6 | ≤0.039 | ≤0.25 | ≤0.25 | ≤0.039 | 4 | ≤0.25 | 0.156 | 0.5 | 0.5 | 0.625 |
| Ge 22 | Germany | not specified | 2019 | Merzen | 10^9 | ≤0.039 | ≤0.25 | ≤0.25 | ≤0.039 | 4 | ≤0.25 | 0.078 | 0.5 | 1 | 0.625 |
| Ge 23 | Germany | joint | 2019 | Lossa | 10^7 | ≤0.039 | ≤0.25 | ≤0.25 | ≤0.039 | 1 | ≤0.25 | 0.039 | 0.25 | 2 | 0.625 |
| Ge 24 | Germany | joint | 2019 | Wennigsen | 10^9 | ≤0.039 | ≤0.25 | ≤0.25 | ≤0.039 | 8 | ≤0.25 | 0.078 | 0.5 | 0.5 | 0.312 |
| Ge 25 | Germany | lung | 2019 | Walsrode | 10^9 | ≤0.039 | 4 | 16 | 0.078 | 1 | 1 | 0.156 | 1 | 4 | 0.312 |
| Hu 1 | Hungary | joint | 2018 | Bácsalmás | 10^9 | ≤0.039 | ≤0.25 | ≤0.25 | ≤0.039 | 4 | ≤0.25 | ≤0.039 | ≤0.125 | 0.25 | 0.156 |
| Hu 2 | Hungary | joint | 2019 | Gerde | 10^9 | ≤0.039 | ≤0.25 | ≤0.25 | ≤0.039 | 4 | ≤0.25 | ≤0.039 | ≤0.125 | 0.5 | 0.156 |
| Hu 3 | Hungary | joint | 2020 | Füle | 10^9 | ≤0.039 | ≤0.25 | 1 | ≤0.039 | 4 | ≤0.25 | 0.078 | 0.25 | 0.5 | 0.156 |
| Hu 4 | Hungary | joint | 2020 | Cegléd | 10^9 | ≤0.039 | ≤0.25 | ≤0.25 | ≤0.039 | 2 | ≤0.25 | ≤0.039 | ≤0.125 | 1 | 0.078 |
| Hu 5 | Hungary | lung | 2021 | Pásztó | 10^8 | ≤0.039 | ≤0.25 | 0.25 | ≤0.039 | 2 | ≤0.25 | 0.039 | 0.25 | 0.5 | 0.312 |
| Hu 6 | Hungary | joint | 2022 | Hódmezővásárhely farm I | 10^9 | ≤0.039 | ≤0.25 | ≤0.25 | ≤0.039 | 16 | ≤0.25 | ≤0.039 | 2 | 1 | 0.039 |
| Hu 7 | Hungary | joint | 2022 | Hódmezővásárhely farm II | 10^8 | ≤0.039 | ≤0.25 | ≤0.25 | ≤0.039 | 16 | ≤0.25 | 0.625 | 2 | 0.5 | 0.156 |
| Hu 8 | Hungary | tonsil | 2023 | Kisújszállás | 10^9 | ≤0.039 | 1 | 2 | ≤0.039 | 64 | ≤0.25 | 0.156 | 0.5 | 1 | 0.078 |
| Hu 9 | Hungary | tonsil | 2023 | Hódmezővásárhely farm III | 10^9 | ≤0.039 | ≤0.25 | ≤0.25 | ≤0.039 | 2 | ≤0.25 | ≤0.039 | ≤0.125 | 1 | 0.078 |
| Hu 10 | Hungary | tonsil | 2023 | Kunszentmárton | 10^10 | ≤0.039 | ≤0.25 | ≤0.25 | ≤0.039 | 4 | ≤0.25 | ≤0.039 | ≤0.125 | 1 | 0.078 |
| Hu 11 | Hungary | tonsil | 2023 | Csemő | 10^8 | ≤0.039 | ≤0.25 | ≤0.25 | ≤0.039 | >64 | ≤0.25 | 0.312 | 2 | 1 | 0.312 |
| Hu 12 | Hungary | tonsil | 2023 | Komárom | 10^8 | ≤0.039 | 1 | 2 | ≤0.039 | 32 | ≤0.25 | 0.312 | 1 | 1 | 0.312 |
| Hu 13 | Hungary | tonsil | 2023 | Bábolna | 10^8 | ≤0.039 | ≤0.25 | 1 | ≤0.039 | 8 | ≤0.25 | 0.156 | 1 | 2 | 0.078 |
| Hu 14 | Hungary | tonsil | 2023 | Ács | 10^9 | ≤0.039 | 0.25 | 1 | ≤0.039 | 8 | ≤0.25 | 0.078 | 0.5 | 1 | 0.078 |
| Hu 15 | Hungary | tonsil | 2023 | Nyárád | 10^9 | ≤0.039 | ≤0.25 | ≤0.25 | ≤0.039 | 32 | ≤0.25 | 0.156 | 0.5 | 1 | 0.312 |
| Hu 16 | Hungary | tonsil | 2023 | Kajánújfalu | 10^9 | ≤0.039 | 0.25 | 1 | ≤0.039 | 16 | ≤0.25 | 0.156 | 0.5 | 0.25 | 0.156 |
| Hu 17 | Hungary | tonsil | 2023 | Bolhás | 10^9 | ≤0.039 | ≤0.25 | 1 | ≤0.039 | 32 | ≤0.25 | 0.156 | 0.5 | 1 | 0.156 |
| Hu 18 | Hungary | tonsil | 2023 | Magyarhomorog | 10^9 | ≤0.039 | ≤0.25 | ≤0.25 | ≤0.039 | 16 | ≤0.25 | 0.156 | 0.5 | 1 | 0.156 |
| Hu 19 | Hungary | tonsil | 2023 | Tomajmonostora | 10^9 | ≤0.039 | ≤0.25 | 1 | ≤0.039 | 8 | ≤0.25 | 0.312 | 2 | 2 | 0.078 |
| Hu 20 | Hungary | tonsil | 2023 | Rácalmás | 10^9 | ≤0.039 | ≤0.25 | 2 | ≤0.039 | 2 | ≤0.25 | 0.156 | 0.5 | 1 | 0.156 |
| Hu 21 | Hungary | joint | 2023 | Hajdúböszörmény | 10^9 | ≤0.039 | ≤0.25 | ≤0.25 | ≤0.039 | 1 | ≤0.25 | ≤0.039 | 0.25 | 1 | 0.625 |
| It 1 | Italy | lung | 2018 | Bolzano | 10^8 | ≤0.039 | 0.5 | 2 | ≤0.039 | 8 | ≤0.25 | 0.078 | 0.5 | ≤0,125 | 0.156 |
| It 2 | Italy | BAL | 2018 | Pordenone I | 10^8 | ≤0.039 | ≤0.25 | ≤0.25 | ≤0.039 | 4 | ≤0.25 | 0.312 | 2 | ≤0,125 | 0.312 |
| It 3 | Italy | lung | 2018 | Treviso | 10^9 | ≤0.039 | 0.5 | ≤0.25 | ≤0.039 | 1 | ≤0.25 | 0.078 | 0.5 | 1 | 0.625 |
| It 4 | Italy | BAL | 2018 | Pordenone II | 10^8 | ≤0.039 | ≤0.25 | ≤0.25 | ≤0.039 | 4 | ≤0.25 | 0.078 | ≤0.125 | 1 | 0.625 |
| It 5 | Italy | lung | 2020 | Vicenza | 10^8 | ≤0.039 | ≤0.25 | ≤0.25 | ≤0.039 | ≤0.25 | ≤0.25 | ≤0.039 | ≤0.125 | 0.5 | 0.312 |
| It 6 | Italy | joint | 2020 | Pordenone III | 10^8 | ≤0.039 | 1 | 4 | ≤0.039 | 16 | ≤0.25 | 0.312 | 1 | 2 | 0.156 |
| It 7 | Italy | pericardium | 2020 | Pordenone IV | 10^8 | ≤0.039 | ≤0.25 | 1 | ≤0.039 | 16 | ≤0.25 | 0.312 | 2 | 2 | 0.156 |
| It 8 | Italy | lung | 2022 | Verona I | 10^9 | ≤0.039 | 1 | ≤0.25 | ≤0.039 | 1 | ≤0.25 | 0.625 | 2 | 2 | 0.312 |
| It 9 | Italy | joint | 2022 | Udine I | 10^8 | ≤0.039 | ≤0.25 | ≤0.25 | ≤0.039 | 8 | ≤0.25 | 0.312 | 1 | 0.5 | 0.312 |
| It 10 | Italy | lung | 2022 | Verona II | 10^9 | ≤0.039 | ≤0.25 | ≤0.25 | ≤0.039 | 32 | ≤0.25 | 2.5 | ≤0.125 | 0.5 | 0.625 |
| It 11 | Italy | lung | 2022 | Verona III | 10^8 | ≤0.039 | 0.25 | 1 | ≤0.039 | 8 | ≤0.25 | 0.156 | 0.5 | 1 | 0.156 |
| It 12 | Italy | joint | 2022 | Mantua | 10^7 | ≤0.039 | ≤0.25 | ≤0.25 | ≤0.039 | 1 | ≤0.25 | 0.078 | 0.125 | 0.25 | 0.312 |
| It 13 | Italy | tonsils | 2023 | Cremona I | 10^9 | ≤0.039 | ≤0.25 | ≤0.25 | ≤0.039 | >64 | ≤0.25 | 0.156 | 1 | 2 | 0.312 |
| It 14 | Italy | tonsils | 2023 | no data | 10^8 | ≤0.039 | 1 | 2 | ≤0.039 | 32 | 0.5 | 0.156 | 1 | 1 | 0.312 |
| It 15 | Italy | tonsils | 2023 | Mantova | 10^9 | ≤0.039 | ≤0.25 | ≤0.25 | ≤0.039 | 64 | ≤0.25 | 0.312 | 2 | 2 | 0.078 |
| It 16 | Italy | tonsils | 2023 | Udine II | 10^8 | ≤0.039 | ≤0.25 | ≤0.25 | ≤0.039 | >64 | ≤0.25 | 0.625 | 4 | 2 | 0.156 |
| It 17 | Italy | tonsils | 2023 | Pordenone V | 10^9 | ≤0.039 | ≤0.25 | ≤0.25 | ≤0.039 | 32 | ≤0.25 | 0.156 | 1 | 1 | 0.156 |
| It 18 | Italy | tonsils | 2023 | Bergamo | 10^8 | ≤0.039 | ≤0.25 | ≤0.25 | ≤0.039 | 16 | ≤0.25 | 0.312 | 2 | 1 | 0.156 |
| It 19 | Italy | tonsils | 2023 | Verona IV | 10^9 | ≤0.039 | ≤0.25 | 1 | ≤0.039 | 32 | ≤0.25 | 0.312 | 2 | 0.5 | 0.312 |
| It 20 | Italy | tonsils | 2023 | Cremona II | 10^9 | ≤0.039 | ≤0.25 | ≤0.25 | ≤0.039 | 4 | ≤0.25 | ≤0,039 | 1 | 0.5 | 0.312 |

*BAL-broncho alveolar lavage
**Province in case of the Italian isolates
***CCU-colour changing units
