# Supplementary material for: Antimicrobial susceptibility profiles of Mycoplasma hyosynoviae strains isolated from five European countries between 2018 and 2023
Source: Sci Rep. 2025 Jan 7;15:1243. doi: 10.1038/s41598-024-85052-1 (PMC11707295; doi:10.1038/s41598-024-85052-1)
Supplement: Supplementary file 4 — Supplementary Information 4. [file 41598_2024_85052_MOESM4_ESM.pdf]

Supplementary table 2

Repeats	Tiamulin	Enrofloxacin	Doxycycline	Oxytetracycline	Tylvalosin	Tylosin	Tilmicosin	Tulathromycin*	Lincomycin	Florfenicol*
1	≤0.039	0.312	0.078	0.5	≤0.039	≤0.25	≤0.25	16	≤0.25	0.25
2	≤0.039	0.312	0.156	0.5	≤0.039	≤0.25	≤0.25	16	≤0.25	0.25
3	≤0.039	0.312	0.078	0.5	≤0.039	≤0.25	≤0.25	8	≤0.25	0.25
4	≤0.039	0.312	0.039	0.125	≤0.039	≤0.25	≤0.25	4	≤0.25	0.25
5	≤0.039	0.312	0.039	0.125	≤0.039	≤0.25	≤0.25	4	≤0.25	0.5
6	≤0.039	0.312	0.078	0.5	≤0.039	≤0.25	≤0.25	4	≤0.25	1
7	≤0.039	0.312	0.039	0.125	≤0.039	≤0.25	≤0.25	2	≤0.25	
8	≤0.039	0.312	0.078	0.5	≤0.039	≤0.25	≤0.25	8	≤0.25	
9	≤0.039	0.625	0.156	1	≤0.039	≤0.25	≤0.25	16	≤0.25	
10	≤0.039	0.312	0.039	0.5	≤0.039	≤0.25	≤0.25	8	≤0.25	
11	≤0.039	0.312	0.039	1	≤0.039	≤0.25	≤0.25	32	≤0.25	

*Minimal inhibitory concentrations (MIC) that differ greater than one dilution-step from the median MIC are highlighted red.
