# Supplementary material for: Antimicrobial susceptibility profiles of Mycoplasma hyosynoviae strains isolated from five European countries between 2018 and 2023
Source: Sci Rep. 2025 Jan 7;15:1243. doi: 10.1038/s41598-024-85052-1 (PMC11707295; doi:10.1038/s41598-024-85052-1)
Supplement: Supplementary file 5 — Supplementary Information 5. [file 41598_2024_85052_MOESM5_ESM.pdf]

Supplementary table 3

ID	Sample type	Isolation year	Farm/Province*	Allele** *dnaA*	*ftsY*	*fusA*	*gyrB*	*recA*	*rpoB*	*uvrA*	Sequence type**	PubMLST ID
Au1	joint	2021	Ybbsitz	9	2	6	12	9	14	15	38	60
Au2	joint	2022	Weitersfeld	10	18	3	8	9	14	7	52	62
Au3	joint	2020	Thanstetten I	12	17	6	6	1	6	4	37	44
Au8	joint	2019	Leibnitz	15	10	9	8	17	9	14	35	89
Au18	joint	2022	Wettmannstätten	7	21	14	12	6	7	22	57	91
Bl 1	tonsil	2023	no data	10	14	6	8	9	**21**	4	**74**	97
Bl 5	tonsil	2023	no data	**20**	16	6	1	16	14	17	**75**	98
Bl 10	tonsil	2023	no data	17	11	15	8	9	14	11	**81**	99
Bl 14	tonsil	2023	no data	**20**	16	6	1	16	14	17	**75**	100
Bl 18	tonsil	2023	no data	15	5	6	12	9	**22**	7	**76**	101
Ge 1	joint	2020	Neustadt am Rübenberge	2	3	6	11	6	1	15	**77**	102
Ge 4	blood	2022	Donauwörth	10	14	6	12	9	14	15	**78**	103
Ge 12	blood	2020	Fürstenau	18	13	14	1	1	**23**	17	**79**	104
Ge 20	joint	2022	Beckum	**21**	13	4	1	1	9	7	**80**	105
Ge 24	joint	2019	Wennigsen	**27**	5	**21**	1	14	**26**	**25**	**91**	116
Hu 1	joint	2018	Bácsalmás	1	1	1	1	1	1	1	1	106
Hu 5	lung	2021	Pásztó	2	**24**	6	**16**	9	9	17	**82**	107
Hu 10	tonsil	2023	Kunszentmárton	2	**25**	13	12	9	14	15	**83**	108
Hu 15	tonsil	2023	Nyárád	18	3	13	12	9	14	17	**84**	109
Hu 21	joint	2023	Hajdúböszörmény	2	**26**	6	10	18	11	14	**85**	110
It 1	lung	2018	Bolzano	14	16	6	8	6	**24**	17	**86**	111
It 6	joint	2020	Pordenone III	11	**27**	6	11	17	**25**	10	**87**	112
It 9	joint	2022	Udine I	18	5	9	12	2	1	**24**	**88**	113
It 15	tonsils	2023	Mantova	**25**	15	13	12	9	14	1	**89**	114
It 20	tonsils	2023	Cremona II	**26**	**28**	9	8	9	9	1	**90**	115

*Province in case of the Italian isolates
**New allele types and sequence types are highlighted by red and bold numbers
