# Supplementary material for: Antimicrobial susceptibility profiles of Mycoplasma hyosynoviae strains isolated from five European countries between 2018 and 2023
Source: Sci Rep. 2025 Jan 7;15:1243. doi: 10.1038/s41598-024-85052-1 (PMC11707295; doi:10.1038/s41598-024-85052-1)
Supplement: Supplementary file 6 — Supplementary Information 6. [file 41598_2024_85052_MOESM6_ESM.pdf]

Supplementary table 4

	Estimate	Std. Error	Z-value	p-value
Coefficients				
Country_Belgium	-0.18	0.60	-0.29	0.77
Country_Germany	1.33	0.58	2.29	0.02
Country_Hungary	-2.65	0.67	-3.98	<0.01
Country_Italy	-0.39	0.61	-0.65	0.52
Threshold coefficients				
threshold.1	-6.03	0.70	-8.64	
spacing	2.57	0.24	10.72	
